# Survival impact of primary tumor resection in de novo metastatic breast cancer patients (GEICAM/El Alamo Registry)

**DOI:** 10.1038/s41598-019-55765-9

**Published:** 2019-12-27

**Authors:** Sara Lopez-Tarruella, M. J. Escudero, Marina Pollan, Miguel Martín, Carlos Jara, Begoña Bermejo, Angel Guerrero-Zotano, José García-Saenz, Ana Santaballa, Emilio Alba, Raquel Andrés, Purificación Martínez, Lourdes Calvo, Antonio Fernández, Norberto Batista, Antonio Llombart-Cussac, Antonio Antón, Ainhara Lahuerta, Juan de la Haba, José Manuel López-Vega, E. Carrasco

**Affiliations:** 1 Instituto de Investigación Sanitaria Gregorio Marañón, Universidad Complutense, Madrid, Spain; 2 Centro de Investigación Biomédica en Red de Oncología, CIBERONC-ISCIII, Madrid, Spain; 3 GEICAM, Spanish Breast Cancer Research Group, Madrid, Spain; 4 Instituto de Salud Carlos III (ISCIII), Madrid, Spain; 5 Hospital Universitario Fundación Alcorcón, Universidad Rey Juan Carlos, Madrid, Spain; 6 Hospital Clínico Universitario, Valencia. Biomedical Research Institute INCLIVA, Universidad de Valencia, Valencia, Spain; 7 Instituto Valenciano de Oncología, Valencia, Spain; 8 Servicio de Oncología Médica, Hospital Clínico San Carlos, Instituto de Investigación Sanitaria del Hospital Clínico San Carlos (IdISSC), Madrid, Spain; 9 Hospital Universitario La Fe, Valencia, Spain; 10 Complejo Hospitalario Virgen de la Victoria, Málaga, Spain; 11 Hospital Universitario Lozano Blesa, Zaragoza, Spain; 12 Hospital de Basurto, Bilbao, Spain; 13 Complejo Hospitalario Juan Canalejo, A Coruña, Spain; 14 Complejo Hospitalario de Albacete, Albacete, Spain; 15 Hospital Universitario de Canarias, Sta Cruz de Tenerife, Spain; 16 Hospital Universitario de Lleida Arnau de Vilanova, Lleida, Spain; 17 Hospital General Universitario Miguel Servet, Zaragoza, Spain; 18 Instituto Oncológico de Guipúzcoa, San Sebastián, Spain; 19 Complejo Hospitalario Reina Sofía, Córdoba, Spain; 20 Hospital Universitario Marqués de Valdecilla, Santander, Spain

**Keywords:** Breast cancer, Cancer epidemiology

## Abstract

The debate about surgical resection of primary tumor (PT) in *de novo* metastatic breast cancer (MBC) patients persists. We explored this approach’s outcomes in patients included in a retrospective registry, named *El Álamo*, of breast cancer patients diagnosed in Spain (1990–2001). In this analysis we only included *de novo* MBC patients, 1415 of whom met the study’s criteria. Descriptive, Kaplan-Meier and Cox regression analyses were carried out. Median age was 63.1 years, 49.2% of patients had single-organ metastasis (skin/soft tissue [16.3%], bone [33.8%], or viscera [48.3%]). PT surgery (S) was performed in 44.5% of the cases. S-group patients were younger, had smaller tumors, higher prevalence of bone and oligometastatic disease, and lower prevalence of visceral involvement. With a median follow-up of 23.3 months, overall survival (OS) was 39.6 versus 22.4 months (HR = 0.59, p < 0.0001) in the S- and non-S groups, respectively. The S-group OS benefit remained statistically and clinically significant regardless of metastatic location, histological type, histological grade, hormone receptor status and tumor size. PT surgery (versus no surgery) was associated with an OS benefit suggesting that loco-regional PT control may be considered in selected MBC patients. Data from randomized controlled trials are of utmost importance to confirm these results.

## Introduction

In western countries metastatic breast cancer (MBC) at first diagnosis is characterized by incidence rates ranging between 4% and 10%, low rates of long-term survival, and estimates of life expectancy between 18 and 24 months^1,2^. Thus, the consensus management of patients presenting MBC has been palliative, focused on prolonging survival and improving quality of life^2^. Although the impact of new treatment strategies on response rates is clinically meaningful, benefits of new treatments on overall survival (OS) are harder to attain in the metastatic setting^3–5^. Nevertheless, survival gains for MBC patients have been reported^6–8^, suggesting that new therapeutic strategies could potentially improve the clinical practice and management of MBC patients. In addition, breakthroughs in diagnostic technology (i.e., PET/TC) now allow the detection of early and asymptomatic metastatic disease. The lower tumor burden in limited stage IV disease makes it potentially more responsive to treatment^9–11^.

Because of these advancements in diagnostics and therapies, clinicians are increasingly acknowledging that MBC patient survival prospects are improving^12–14^ and even challenging the paradigm of incurability in selected cases of *de novo* MBC patients^15–17^, particularly those with oligometastatic disease^18–20^. Stemming from the “long-term MBC survivor profile” concept, the role of local therapies is being re-examined beyond its traditional palliative intent and into a potentially curative one^21^.

Retrospective reports and meta-analyses suggest a survival benefit associated with surgery of the primary tumor (PT) in MBC patients, especially when surgical margin is tumor free^22–24^. Thus, surgery may be considered an option in the algorithm for management of selected MBC patients besides the generally accepted hygienic indication as prevention of local complications (infection, ulceration or bleeding). The achievement of locoregional control of the tumor has been tried through surgery but radiotherapy’s role has also been studied in this setting^25,26^. Due to the retrospective nature of these studies, reasonable concerns emerged regarding the consistency of the available clinical data^27,28^. Further, recent work, a U.S.-based prospective registry (TBCRC013) and two randomized clinical trials from Turkey and India^29–31^, have reported conflicting results regarding the impact of PT locoregional treatment on OS. Therefore, international guidelines recommend PT surgery only in selected patients while awaiting for the final results of ongoing clinical trials (ECOG 2108/NCT0124800, SUBMIT/NCT01392586, JCOG 1017/UMIN000005586 y ABCSG 28/POSYTIVE/NCT01015625)^32^.

The biological mechanisms underlying the aforementioned results involve the crosstalk among the PT and metastatic foci, PT immunosuppressive effects, and/or the prevention of metastatic re-seeding from the primary source. Yet, the surgical procedure itself may have negative impacts such as a transient inflammation, turning on the “angiogenic switch” in the wound healing process, and a temporary immunosuppressive state which, subsequently, might imbalance microenvironment-tumor interactions eventually triggering the metastatic cascade^33–37^.

The main aim of this work was to study the prognostic value of PT surgical excision, in the presence of other well-known prognostic factors, in MBC patients with OS as the outcome of interest. The objective of the analysis was to clarify the role of a locoregional approach to the PT in *de novo* MBC patients in a real-world retrospective series. Our ultimate purpose is to evaluate this treatment as a valid option to be included in the national clinical protocol for this patient population. All patients were diagnosed and treated in various Spanish health care institutions included under the umbrella of GEICAM Spanish Breast Cancer Group.

## Patients and Methods

*El Álamo* study is a cooperative epidemiologic initiative conducted by GEICAM in order to characterize breast cancer (BC) cases diagnosed in Spain. *El Álamo* is a retrospective registry of patients diagnosed with BC between 1990 and 2001 across 56 Spanish hospitals. The patterns of BC presentation (tumor and host characteristics), the different treatments received, and the clinical evolution of the disease were described and structured in 3 cohorts collected in consecutive 4-year intervals as follows: *El Álamo I* (1990–1993, 4551 patients, closed by 2000), *El Álamo II* (1994–1997, 10849 patients, closed by 2003) and *El Álamo III* (1998–2001, 10675 patients, closed by 2007). This database is estimated to comprise approximately 15% of the newly diagnosed BC cases in Spain within this period and to reflect clinical practice in the country at each time point. Participating investigators, which included all the medical oncology department staff members of the participating hospitals, were required to include all BC patients seen in those institutions over the entire annual study period.

Only women with invasive *de novo* MBC were included in the current analysis. Patients with stage I–III at initial diagnosis and later recurrence, with secondary tumors, with fewer than 2 months’ follow-up at the same institution, and with missing data regarding their PT surgery were excluded. The criteria to perform PT surgery were defined by institutional guidelines as per investigator discretion.

A total of 39 variables including demographic (age and menopausal status), primary tumor staging (TNM classification), pathology (hormonal receptor status, grade and histological type), and local plus systemic treatment data were selected for this analysis.

Data were collected following the requirements of the Spanish legislation for privacy data protection at the time: the Spanish Organic Law of December 13^th^, 1990 of Personal Data Protection. Observational retrospective studies based on patient charts required neither patient informed consent nor ethical committee approval. We submitted our study to a present-day ethical committee, Hospital Universitario Parc Tauli at Barcelona, which reviewed and confirmed the procedures followed guaranteed patient’s data confidentiality and were in compliance with the rules established at that time.

## Statistical Analysis

Categorical variables were expressed as the absolute and relative frequencies. Continuous data were expressed as central tendency and dispersion measures (mean, median, trends, range). Baseline differences in categorical variables were assessed by the Chi Square test (Χ^2^) or the Fisher exact test when appropriate. OS was defined as the time from the year of diagnosis to death from any cause or to last follow-up visit. OS curves were estimated using the Kaplan-Meier method and survival functions among the different subgroups were compared with the log-rank exact test. The impact of our main variable of interest (PT surgery) and clinically relevant variables (metastatic location, patient’s menopausal status, and tumor’s characteristics including histological type, histological grade, HER2 status, hormone receptor status, Ki67, expression level of p53, BC subtype, and tumor size) on OS was estimated using Cox proportional hazards regression models. The proportional Hazards assumption was checked using the test based on the correlation coefficient between survival time and the scaled Schoenfeld residuals. All tests were performed using an alpha of 0.05.

Analyses were performed using the program R and the Statistical Analysis System (SAS) package (Enterprise Guide 5.1 software, SAS Institute Inc., Cary, NC, USA).

## Results

### Descriptive analyses

**Figure 1:**
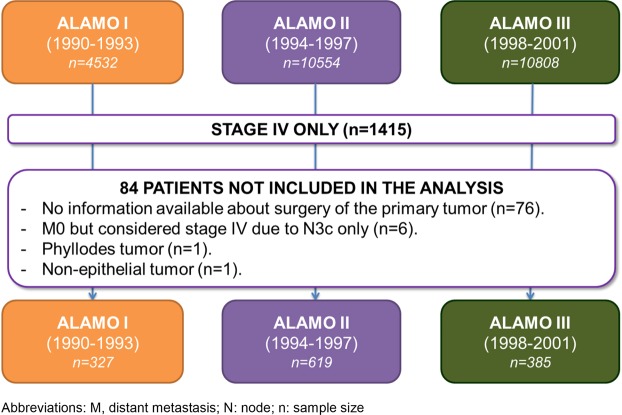
Patient selection flowchart.

We selected 1415 (5.5%) patients with *de novo* MBC from the database *El Álamo*. Of these, 1331 patients (327 patients from *El Álamo I*, 619 patients from *El Álamo II*, and 385 patients from *El Álamo III*) met the eligibility criteria to be included in this analysis (Fig. 1). Table 1 shows patient and tumor characteristics, and treatment received. Median age was 63.1 years (range: 21.6–96.0) and 49.2% of them had single-organ metastasis. The metastatic disease was anatomically distributed as follows: skin/soft tissue (16.3%), bone (33.8%), viscera (48.3%) and unknown/unavailable (8.6%).

**Table 1:** Patient characteristics by surgical management of primary tumor for the entire group *de novo* MBC patients from *El Álamo* registry.

Category	Surgery N = 592 (%)	Non-Surgery N = 739 (%)	p value*
**Age (years)**			**<0.0001**
≤44	115 (19.4)	87 (11.8)	
45–64	240 (40.5)	283 (38.3)	
≥65	236 (39.9)	367 (49.6)	
NA	1 (0.2)	2 (0.3)	
**Menopausal status**			**0.0001**
Postmenopausal	419 (70.8)	589 (79.7)	
Premenopausal	168 (28.4)	144 (19.5)	
NA	5 (0.8)	6 (0.8)	
**Tumor size (TNM)**			<0.0001**
T0	5 (0.8)	5 (0.7)	
T1	63 (10.6)	39 (5.3)	
T2	223 (37.8)	124 (16.8)	
T3	60 (10.1)	84 (11.4)	
T4	212 (35.8)	372 (50.2)	
NA	29 (4.9)	115 (15.6)	
**Histological type**			<0.0001***
Ductal	426 (72)	322 (43.6)	
Lobular	42 (7.1)	48 (6.5)	
Mucinous (colloid)	5 (0.8)	2 (0.3)	
Mixed	8 (1.4)	2 (0.3)	
Tubular	2 (0.3)	2 (0.3)	
Medullary	1 (0.2)	1 (0.1)	
Papillary	—	1 (0.1)	
Adenocarcinoma (NS)	16 (2.7)	72 (9.7)	
Others (non-adenocarcinoma or NA)	92 (15.5)	289 (39.1)	
**Histological grade**			**0.2734**
GI	31 (5.2)	20 (2.7)	
GII	163 (27.5)	63 (8.5)	
GIII	138 (23.3)	57 (7.7)	
NA	260 (44)	599 (81.1)	
**Hormone Receptor status**			**0.4216**
Positive	286 (48.3)	180 (24.4)	
Negative	102 (17.2)	55 (7.4)	
NA	204 (34.5)	504 (68.2)	
**HER2 status**			**0.1243**
Positive	30 (5.1)	22 (3.0)	
Negative	100 (16.9)	44 (6.0)	
NA	462 (78.0)	673 (91.0)	
**Ki67 (cut-off 13%)**			**0.3309**
High (>13%)	24 (4.1)	13 (1.8)	
Low (≤13%)	14 (2.4)	4 (0.5)	
NA	554 (93.5)	722 (97.7)	
**p53**			**0.4370**
Positive	25 (4.2)	7 (0.9)	
Negative	28 (4.7)	12 (1.6)	
NA	539 (91.1)	720 (97.5)	
**BC Subtypes**			**0.0758**
HR positive/HER2 negative	76 (12.8)	27 (3.7)	
HER2positive	30 (5.1)	22 (3)	
Triple Negative	18 (3.0)	5 (0.7)	
NA	468 (79.1)	685 (92.6)	
**Number of organs with metastasis**			**<0.0001**
1	340 (57.3)	315 (42.6)	
2	100 (16.9)	170 (23.0)	
≥3	76 (12.9)	196 (26.5)	
NA	76 (12.9)	58 (7.9)	
**Metastatic location**	N = 528	N = 689	**<0.0001**
Skin/Soft tissue	96 (18.2)	102 (14.8)	
Bone	206 (39.0)	205 (29.8)	
Visceral	213 (40.3)	375 (54.4)	
NA	13 (2.5)	7 (1.0)	

Abbreviations: MBC, metastatic breast cancer; NA, not assessed; T, tumor size; NS, non-specific; G, grade; BC, breast cancer; HR, hormone receptor; HER2, human epidermal growth factor receptor 2.

*p-value calculated not considering NA or Other data; **Patients with T0 and T1 tumors were combined; ***ductal versus others.

**Figure 2:**
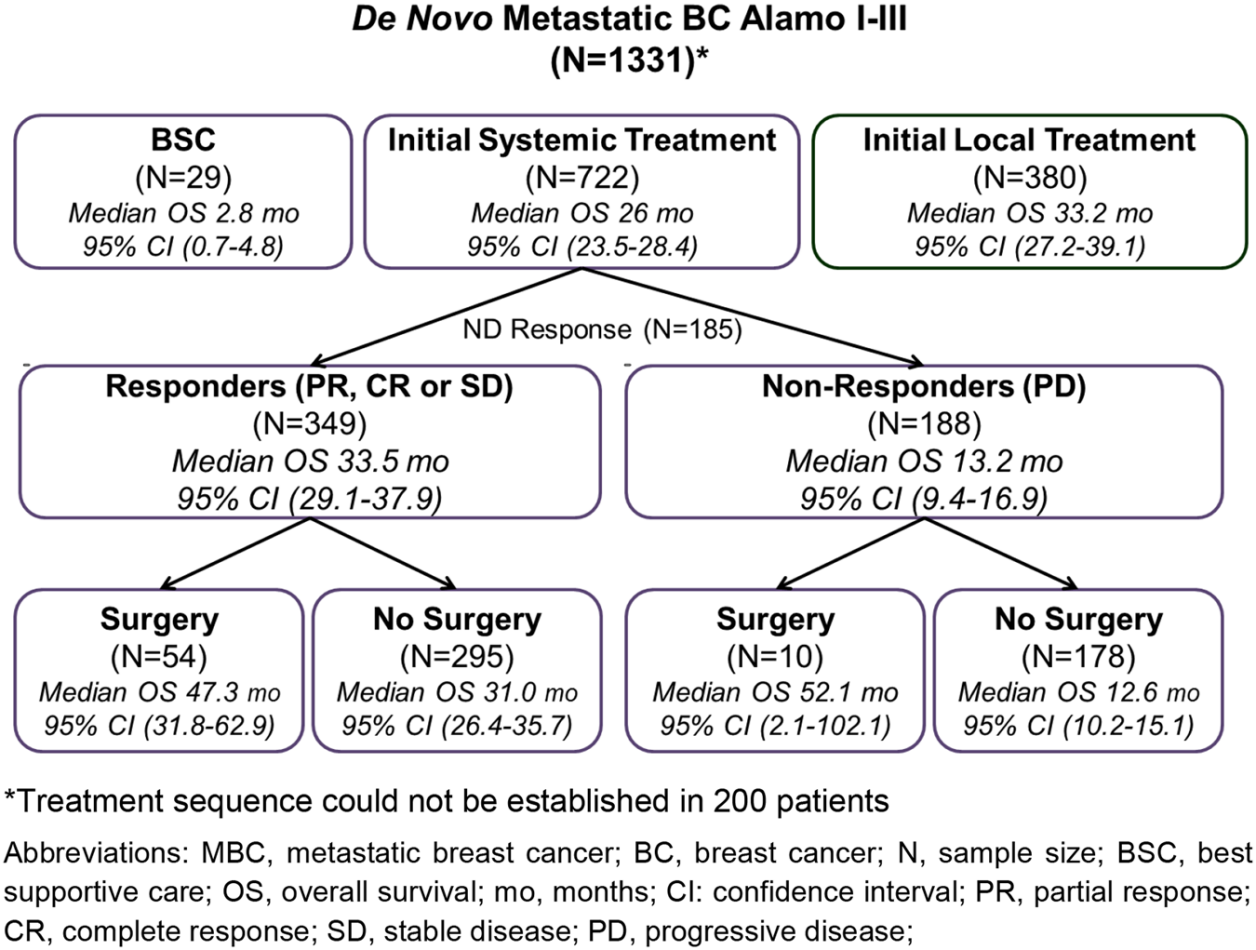
Local and systemic treatments administered to *de novo* MBC patients and corresponding survival outcomes from El Álamo registry.

Initial local treatment was the choice for 380 (28.5%) patients (358 surgery and 22 radiotherapy), 722 (54.2%) patients received initial systemic therapy (480 chemotherapy [CT], 214 endocrine treatment [ET] and 28 both CT and ET), 29 (2.2%) received best supportive care and for the rest of patients the treatment sequence could not be established (Fig. 2).

PT surgery, at any time of the patient evolution, was performed in 44.5% (N = 592) patients (512 radical procedures [86.5%], 46 palliative procedures [7.8%], and 34 unknown [5.7%]). Out of the 592, 427 (72.1%) patients underwent axillary lymph node dissection. Compared to women in the non-surgery group (non-S), women in the surgery group (S) were younger (19.4% of the S group versus 11.8% of the non-S group were ≤44 years-old, respectively), a higher percentage presented with oligometastatic disease (defined as metastases limited to a single organ) than not (57.4% versus 42.6%, respectively), were more likely to have smaller (i.e. ≤T2) tumors (49.2% versus 22.7%, respectively), and were less likely to present visceral disease (40.3% versus 54.4%) but more likely to present bone metastases (39% versus 29.8%, respectively).

### Survival analysis

**Figure 3:**
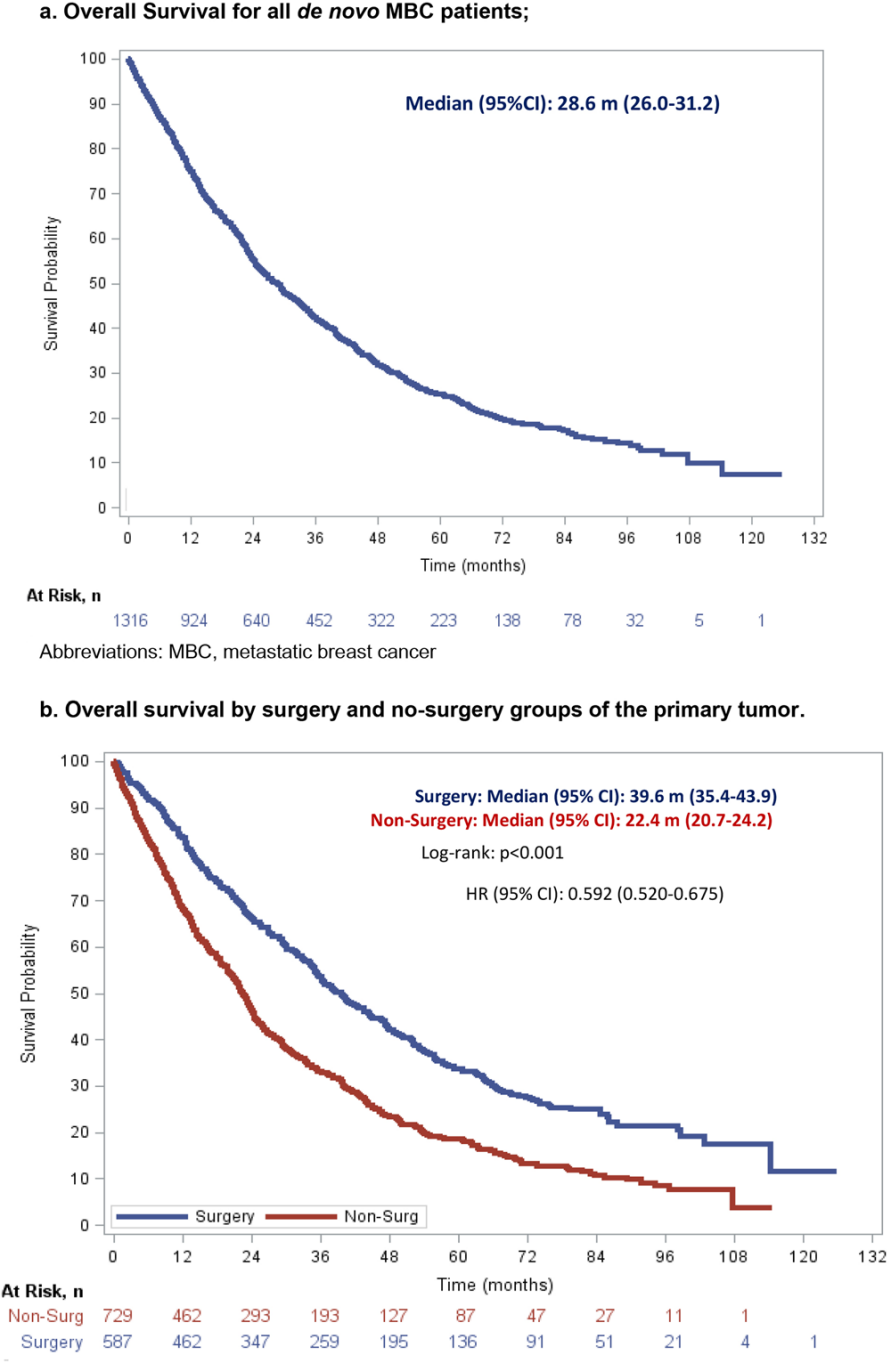
Overall survival for *El Álamo* registry patients. (**a**) Overall Survival for all *de novo* metastatic breast cancer patients; (**b**) Overall survival by surgery and no-surgery groups of the primary tumor.

With a median follow-up of 23.3 months, median OS was 28.6 months (95% Confidence Interval [CI], 26.0–31.2). At 1 year, 75.3% of patients (95%CI, 72.9–77.6) were alive, 42.6% remained alive at 3 years (95%CI, 39.8–45.4), and only 25.4% (95%CI, 22.8–28) were alive at 5 years (Fig. 3a).

Median OS in the S group was 39.6 months (95%CI, 35.4–43.9) compared to 22.4 months (95%CI, 20.7–24.2) in the non-S group (Hazards Ratio [HR] 0.59, 95%CI, 0.52–0.68; p < 0.0001). At 1 year, 83.6% of patients (95%CI, 80.5–86.6) were alive in the S group compared to 68.6% (95%CI, 65.2–72.0) in the non-S group. This S group survival advantage was maintained in the following years with 53.7% of patients (95%CI, 49.4–57.9) being alive in the S group versus 33.5% (95% CI, 29.9–37.2) in the non-S group at 3 years. Finally, at 5 years, survivors decreased to 33.5% of patients (95%CI, 29.3–37.8) in the S group versus 18.7% (95% CI, 15.5–21.9) in the non-S group (Fig. 3b).

**Figure 4:**
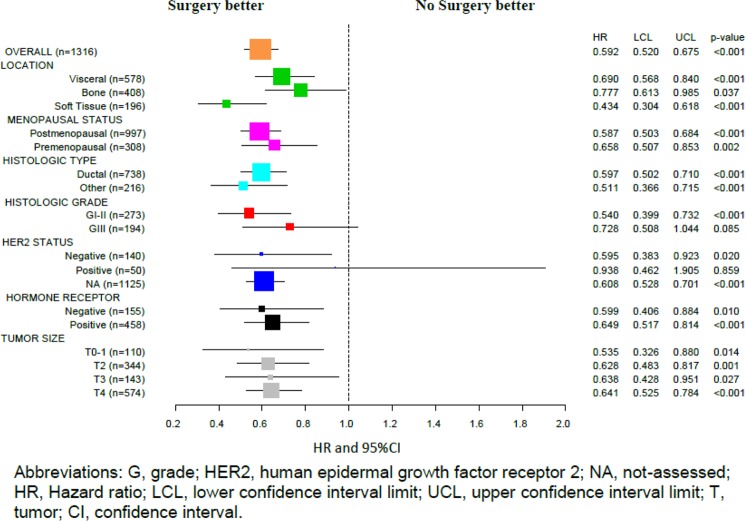
Subgroup analysis of overall survival from *El Álamo* registry.

Subgroup OS analysis also showed a consistent benefit among the S group across all selected categories of tumor characteristics (Fig. 4).

#### Univariate analysis for the entire de novo MBC group in the El Álamo registry

PT surgery, metastatic location, histological type, histological grade, hormone receptor status, BC subtype, and tumor size were statistically significant prognostic factors for OS in the univariate Cox analysis. On the other hand, menopausal status (p = 0.144), HER2 status (p = 0.061), Ki67 (p = 0.972), and p53 (p = 0.504) failed to have a significant impact on OS (Table 2).

**Table 2:** Univariate analysis of overall survival for the entire group of *de novo* MBC patients from *El Álamo* registry.

Variable	N	Events	Censored	Hazard Ratio (95%CI)	p-value
**Surgery**					**<0,0001**
Yes	587	384	203	0.592 (0.520–0.675)	
No	729	560	169	Ref.	
**Metastatic location**					**<0.0001**
Visceral	578	455	123	Ref.	
Bone (if non visceral)	408	276	132	0.606 (0.521–0.704)	<0.0001
Soft tissue (if non visceral non bone)	196	129	67	0.568 (0.466–0.691)	<0.0001
**Menopausal status**					0.144
Premenopausal	308	230	78	Ref.	
Postmenopausal	997	707	290	1.117 (0.963–1.297)	
**Histological type**					**0.006**
Ductal	738	516	222	Ref.	
Other	216	163	53	1.282 (1.074–1.529)	
**Histological grade**					**0.001**
G I-II	273	184	89	0.676 (0.542–0.844)	
G III	194	139	55	Ref.	
**HER2 status**					**0.061**
Positive	51	35	16	Ref.	
Negative	140	85	55	0.685 (0.461–1.017)	
**Hormone receptor status**					**0.001**
Positive	459	309	150	0.686 (0.550–0.854)	
Negative	155	108	47	Ref.	
**Ki67**					0.972
0–13	18	13	5	0.988 (0.485–2.009)	
>13	36	19	17	Ref.	
**P53**					0.504
Positive	31	17	14	Ref.	
Negative	40	27	13	0.811 (0.439–1.499)	
***El Álamo*** **cohort**					**0.003**
*El Álamo I*	327	248	79	Ref.	
*El Álamo II*	611	438	173	0.882 (0.755–1.031)	
*El Álamo III*	378	258	120	0.742 (0.623–0.884)	
**BC Subtype**					**0.039**
TN	23	12	11	Ref.	
HER2positive	51	32	16	1.523 (0.790–2.937)	
HR positive/HER2 negative	99	57	42	0.881 (0.473–1.642)	
**Tumor size (TNM)**					**<0.0001**
T0	10	5	5	0.322 (0.133–	
T1	101	58	43	0.779)	
T2	344	236	108	0.549 (0.417-	
T3	143	103	40	0.723)	
T4	574	437	137	0.740 (0.631–0.867)	
				0.886 (0.715–1.099)	
				Ref.	
**Number of metastases**					**<0.0001**
Single-organ metastasis	650	443	207	0.703 (0.615–0.804)	
Multiple metastases	532	417	115	Ref.	

Abbreviations: MBC, metastatic breast cancer; CI: Confidence interval; G, grade; BC, breast cancer; TN, triple negative; HER2, human epidermal growth factor receptor 2; HR, hormone receptor; T, tumor size.

#### Multivariate analysis for the entire de novo MBC group in the El Álamo registry

Adjusting for all relevant clinical variables in a multivariate model (except BC subtypes and HER2 due to their small sample sizes), the fully-adjusted HR for PT surgery was 0.685 (95%CI, 0.591–0.793; p < 0.001). All the following variables were found to be statistically significant in the final multivariate Cox regression analysis: metastatic location, histological type, histological grade, hormone receptor status and tumor size (Table 3). Given that HRs for metastatic location and histological type did not meet the proportional hazard assumption, we carried out a sensitivity analysis stratifying the cohort according to these two factors. The HR for PT surgery was virtually unchanged (0.685 in the final model versus 0.684 in the stratified model).

**Table 3:** Multivariate analysis for overall survival for the entire *de novo* MBC patients from *El Álamo* registry^*^.

Variable	N	Hazard Ratio	95%CI	p-value
**Surgery**				**<0,001**
Yes	587	0.685	(0.591–0.793)	
No	729	Ref.		
**Metastatic location**				**<0,001**
Visceral	578	Ref.		
Bone (if non visceral)	408	0.674	(0.578–0.787)	
Soft tissue (if non bone non visceral)	196	0.554	(0.452–0.680)	
NA	134	0.697	(0.547–0.886)	
**Histological type**				**0,003**
Ductal	738	Ref.		
Other	216	1.249	(1.041–1.498)	
NA	362	0.863	(0.725–1.028)	
**Histological Grade**				**0.009**
G I-II	273	0.721	(0.576–0.904)	
G III	194	Ref.		
NA	849	0.755	(0.613–0.929)	
**Hormone Receptor status**				**<0.001**
Positive	459	0.676	(0.539–0.848)	
Negative	155	Ref.		
NA	702	0.983	(0.780–1.241)	
***El Álamo*** **cohort**				**0,213**
*El Álamo I*	327	Ref.		
*El Álamo II*	611	0.940	(0.798–1.108)	
*El Álamo III*	378	0.839	(0.687–1.025)	
**Tumor Size (TNM)**				**0.048**
T0	10	0.358	(0.147–0.873)	
T1	101	0.708	(0.534–0.939)	
T2	344	0.887	(0.753–1.046)	
T3	143	0.930	(0.748–1.156)	
T4	574	Ref.		
NA	144	0.967	(0.779–1.200)	

Abbreviations: MBC, metastatic breast cancer; CI: confidence interval, NA not-assessed; G, grade; T, tumor size.

^*^Even though the variable “Number of metastases” was statistically significant in the univariate analysis, it was not included in the multivariate model because of a multicollinearity problem with the variable “metastatic location”.

#### Restricted multivariate analysis (low risk)

Due to the observational nature of the *El Álamo* study and in order to avoid a possible confusion due to indication bias, we performed an exploratory sensitivity analysis for patients with extreme favorable prognostic features. The definition of this “low risk” population comprised patients with small tumor size (≤T2) and oligometastatic disease. We repeated the multivariate Cox regression analysis in this particular subgroup of “low risk” *de novo* MBC patients (N = 252).

The median OS for the low-risk subgroup was 40.4 months (95%CI, 33.2–47.7) with 53.2% (95%CI, 46.7–59.7%) of patients being alive at 3 years. The fully adjusted HR for PT surgery was 0.742 (95%CI, 0.518–1.063; p = 0.103), i.e., it failed to be a prognostic factor for this low-risk subgroup. However, metastatic location, histological type, and histological grade remained statistically significant predictors of survival. In addition, year of diagnosis (i.e., *El Álamo* cohort) was also a significant prognostic factor in this patient subgroup.

#### Subgroup analysis based on the approach for the primary tumor

Table 4 shows a subgroup analysis including metastatic location, histological type, histological grade, hormone receptor status, *El Álamo* cohort, BC subtype, tumor size, and number of metastases. Median OS was greater in patients with PT surgical excision regardless of metastatic location (visceral, bone, skin/soft tissue), histological type, hormone receptor status, *El Álamo* cohort, tumor size, and whether they had single or multiple metastases. However, this benefit failed to reach statistical significance when evaluating it by BC subtypes (due to small sample sizes) or for patients with histological grade III tumors.

**Table 4:** Subgroup analysis of overall survival in the surgery and non-surgery groups from El Álamo registry.

Subgroup	Number of events/patients (%)		Median OS (months) (95%CI)	
	Non-Surgery	Surgery	Non-Surgery	Surgery
**All**^**a**^	560/729 (77)	384/587 (65)	22.4 (20.7–24.2)	39.6 (35.4–43.9)
**Metastatic location**				
Visceral^a^	300/367 (82)	155/211 (73)	18.1 (14.7–21.4)	27.9 (20.8–35.0)
Bone^b^	140/204 (69)	136/204 (67)	30.6 (25.2–35.9)	42.5 (35.5–49.6)
Skin and Soft tissue^a^	76/101 (75)	53/95 (56)	21.9 (18.1–25.7)	52.1 (37.5–66.8)
**Histological type**				
Ductal^a^	246/316 (78)	270/422 (64)	23.4 (21.2–25.6)	38.7 (33.9–43.6)
Other^a^	113/138 (82)	50/78 (64)	16.0 (10.1–22.0)	37.9 (19.8–56.0)
**Histological grade**				
G I-II^a^	66/82 (80)	118/191 (62)	25.0 (18.2–31.8)	49.7 (40.8–58.6)
G III^c^	43/56 (77)	96/138 (70)	21.2 (12.5–29.9)	29.4 (20.6–38.3)
**Hormone receptor status**				
Positive^a^	130/176 (74)	179/283 (63)	31.8 (26.3–37.4)	46.5 (40.6–52.3)
Negative^d^	44/54 (81)	64/101 (63)	20.8 (10.6–30.9)	23.6 (10.2–36.9)
**El Álamo cohort**				
*El Álamo I*^a^	159/203 (78)	89/124 (72)	21.2 (17.6–24.8)	35.6 (28.1–43.2)
*El Álamo II*^a^	275/357 (77)	163/254 (64)	21.9 (19.6–24.2)	38.0 (32.0–44.0)
*El Álamo III*^e^	126/169 (75)	132/209 (63)	25.3 (17.8–32.7)	43.6 (35.5–51.7)
**BC subtype**				
Triple negative	5/5 (100)	7/18 (39)	40 (0–94.0)	—
HER2 positive (regardless HR status)	12/21 (57)	23/30 (77)	38.3 (16.4–60.2)	41.8 (29.3–54.3)
HR positive and HER2 negative	17/25 (68)	40/74 (54)	39.3 (26.3–52.4)	52.4 (39.9–64.9)
**Tumor size (TNM)**				
T0	2/5 (40)	3/5 (60)	—	59.2 (10.7–107.7)
T1^f^	27/38 (71)	31/63 (49)	23.9 (16.8–30.9)	64.2 (42.4–86.1)
T2^a^	93/123 (76)	143/221 (65)	24.0 (19.2–28.7)	40.0 (32.4–47.6)
T3^g^	62/83 (75)	41/60 (68)	24.2 (16.2–32.3)	41.0 (20.1–61.9)
T4^a^	291/365 (80)	146/209 (70)	21.6 (19.2–24.0)	35.1 (30.4–39.8)
**Number of metastases**				
Single-organ metastasis^a^	228/313 (73)	215/337(64)	24.5 (22.1–26.9)	40.4 (34.5–46.3)
Multiple metastases^e^	287/358 (80)	130/174 (75)	20.8 (17.3–24.2)	32.1 (28.2–36.0)

Abbreviations: OS, overall survival; G, grade; BC, breast cancer; HER2, human epidermal growth factor receptor 2; HR, Hormone receptor; T, tumor size.

^a^p < 0.0001; ^b^p = 0.037; ^c^p = 0.083; ^d^p = 0.009; ^e^p = 0.001; ^f^p = 0.002; ^g^p = 0.026.

## Discussion

The OS benefit observed in *de novo* MBC patients from *El Álamo* registry undergoing PT surgery supports previous findings reported by contemporary population-based registries^14,38–43^. Two meta-analyses found that PT surgery in these patients was independently associated with improved survival, HR of 0.63–0.69^22,23^, ranging from 0.5 to 0.8 in the individual series. However, since other studies failed to detect any benefit from PT surgery^44–46^, findings in this field remain inconclusive. *El Álamo* data yielded a median OS of 39.6 versus 22.4 months (HR = 0.59, p < 0.0001), for the S and non-S group, respectively. These figures are comparable to the most favorable data reported by previous studies.

*De novo* MBC patients represented 5.5% of the entire BC patient population included in the El Álamo registry. PT surgery was performed in 44.5% of them. These results align with previously reported population-based evidence from other European registries^39,41^. In *El Álamo* registry 54.2% of patients received primary systemic therapy and underwent surgery later on.

Although retrospective studies offer valuable information in a very efficient, cost-effective manner, prospective studies are needed to confirm any findings and advance knowledge. For instance, prospective analyses from a US population multicentre registry^29^ recently showed that patients selected for surgery were more likely to have oligometastatic disease and to receive first-line chemotherapy. Among responders, surgery was not associated with improved survival for any BC subtype. Several clinical trials were designed to prospectively address this finding, but results from the two randomized trials already reported failed to provide a satisfactory answer. A Turkish study (MF07–01)^30^ requiring randomization to loco-regional therapy (LRT) before systemic therapy, reported an OS benefit from initial surgery (median OS of 46 versus 37 months in the S and non-S groups, respectively [HR 0.66, p = 0.005]), suggesting a potential benefit mainly in patients with bone-only metastases (supporting our results), in patients with ER positive/HER2 negative tumors, as well as in younger patients. In contrast, the Tata Memorial study^31^ requiring induction systemic therapy prior to randomization, reported no LRT impact on OS in patients who responded to front-line chemotherapy (median OS of 19.2 versus 20.5 months in the LRT versus non-LRT groups [HR 1.04, p = 0.79]). Nonetheless, local progression-free survival (PFS) was significantly better in patients who had LRT (HR 0.16, p < 0.001) in contrast with the significant detriment in distant PFS in patients receiving LRT (HR 1.42, p = 0.012). No differences in survival were detected in the planned subset analysis. In addition, preliminary results from the ABCSG 28 study^47^ comparing initial PT surgery versus initial systemic treatment in only 90 patients (the study was terminated early due to low accrual) failed to find OS benefit for immediate PT surgery (HR 0.69, p = 0.267). Two other studies (JCOG1017 and ECOG2108) testing the value of surgery after primary systemic treatment are currently ongoing. Although it is agreed upon that level 1 evidence, e.g., prospective data from these randomized-controlled trials like these, is highly needed to close this long-lasting debate, it remains difficult to obtain in a timely manner. Moreover, the interpretation of their results should be done carefully from a methodological perspective and contextualized with the available body of real-world evidence^48^.

Our results should be interpreted in the context of the study’s and registry’s limitations. For instance, despite the current evidence of the importance of achieving PT surgery with negative margins for improved outcomes^38,39,49^ this information was not available in *El Álamo* database. Similarly, the database did not address the highly relevant aspect of the impact on survival of PT local radiation therapy, usually studied as part of a multimodal locoregional approach. Thus, whether there is an actual benefit of radiotherapy by itself in this setting, the optimal dose, and fractionation remain unknown^26^ and, thus, cannot be recommended as a standard procedure.

Additionally, because of the observational nature of our series, we performed a multivariate sensitivity analysis in the “low risk” *de novo* MBC patient subgroup. By reducing the heterogeneity of both known and unknown prognostic factors potential biases are reduced. Results showed that the prognostic value of PT excision surgery was preserved overall, strongly suggesting that the response of this “low-risk” subgroup of patients to PT surgery is not different from that of the *de novo* MBC patient population under study.

Further, certain limitations of our data could confound the association between PT surgery and survival, thus affecting how our results inform decision-making algorithms in clinical practice. Specifically, two sources of potential bias should be recognized as selection biases. First, the stage migration phenomenon defining stage IV MBC (TNM-staging classifications changed between 1990 and 2001 in the definition of N3 supraclavicular nodes versus M1); and second, the non-standardized indication of the local procedure which was conducted on a case by case basis, with no pre-defined criteria, based on the physician’s discretion.

Also, the lack of on-site monitoring of data compilation and data quality at the different institutions introduces an additional source of variability. Thus, we consider the amount of missing data for the following variables another study limitation: hormone receptor and HER2 status for the molecular sub-classification of patients, surgical procedures, pathological margin status, and chronological order of administration of loco-regional and systemic therapies.

This study also has important strengths. First, to our knowledge this is the largest series performed in Spain (N = 1331) assessing the role of PT surgery in *de novo* MBC patients’ survival. Second, the wide geographical distribution of cases throughout Spain in the late nineties captures the clinical practices in place at that time to manage the controversial aspect of PT surgery as part of the management of a very small but remarkable group of patients. Clinical practices have changed over the last 10–15 years as new treatments have been added to the therapeutic armamentarium. This progress may limit the translational value of the El Álamo results to current clinical practice, but there are still valuable lessons to be learned from them. To address these limitations El Álamo IV (NCT03210974) registry is currently ongoing covering the 2002–2005 period. Moreover, GEICAM is also running, in a parallel fashion, a multicenter prospective registry in the metastatic disease setting (RegistEM, NCT02819882) which will allow the study of how primary tumors are locally managed in a real clinical setting.

Finally, the prognostic value of PT surgical resection may vary by intrinsic subtype, global burden of disease, and/or characteristics of the de novo MBC patients. The definition of the best timing to perform the local approach remains open in the era of molecularly targeted therapy. This is also the case for the need for radiation therapy, the type of surgery required (with or without axillary resection), or even the possibility of foregoing surgery altogether in favor of full radiation treatment.

## Conclusions

In the *El Álamo* registry local PT surgery was associated with better OS independently of metastatic location, histological type, histological grade, hormone receptor status, and tumor size. These analyses suggest that LRT of PT should be considered as part of the therapeutic strategy for selected patients with advanced disease. Thus, this strategy must be further investigated in randomized controlled trials for *de novo* MBC patients to control for potential bias intrinsically associated with retrospective studies. Pre-clinical models and translational correlative studies are needed to understand the biology behind the effect of LRT to the PT in this setting.

### Ethical approval and inform consent

Data were collected following the requirements of the Spanish legislation for privacy data protection in the considered period. The specific legislation is the Spanish Organic Law of December the 13th, 1990 of Personal Data Protection.

The specific regulation for observational retrospective studies was developed in Spain in 2009 with the December 16th ORDER SAS/3470/2009, which publishes guidelines on post-authorization observational studies for drugs for human use. The ALAMO study was performed before this legislation was in place so the ethical committees in Spain did not review any observational retrospective study before this legislation was developed.

According to the current regulations for observational retrospective studies in force the informed consent of the subjects is not required since: (1) in the process of data collection retrospectively, a secure dissociation procedure is adopted, ensuring that the information handled in the study does not contain personal data and (2) personal interviews are not required nor are biological samples collected from patients. Each patient receives a number identification for their participation in the study.

## Supplementary information

Supplementary Table 1

## Data Availability

Data are available from the corresponding author upon reasonable request.
