# Supplementary material for: Survival impact of primary tumor resection in de novo metastatic breast cancer patients (GEICAM/El Alamo Registry)
Source: Sci Rep. 2019 Dec 27;9:20081. doi: 10.1038/s41598-019-55765-9 (PMC6934456; doi:10.1038/s41598-019-55765-9)
Supplement: Supplementary file 1 — Supplementary Table 1 [file 41598_2019_55765_MOESM1_ESM.pdf]

TITLE PAGE

Survival impact of primary tumor resection in de novo metastatic breast cancer patients (GEICAM/El Alamo Registry)

AUTHORS

*Sara Lopez-Tarruella[1,2], MJ Escudero[3], Marina Pollan[4], Miguel Martín[1,2], Carlos Jara[5], Begoña Bermejo[6,4], Angel Guerrero-Zotano [7], José García-Saenz[8,4], Ana Santaballa[9], Emilio Alba[10,4], Raquel Andrés[11], Purificación Martínez[12], Lourdes Calvo[13], Antonio Fernández[14], Norberto Batista[15], Antonio Llombart-Cussac[16], Antonio Antón[17], Ainhara Lahuerta[18], Juan de la Haba[19,4], José Manuel López-Vega[20], E Carrasco[3]

1 Instituto de Investigación Sanitaria Gregorio Marañón. Universidad Complutense, Madrid; Spain. 2 Centro de Investigación Biomédica en Red de Oncología, CIBERONC-ISCIII; Spain. 3 GEICAM, Spanish Breast Cancer Research Group, Madrid; Spain 4 Instituto de Salud Carlos III (ISCIII), Madrid; Spain. 5 Hospital Universitario Fundación Alcorcón, Universidad Rey Juan Carlos, Madrid; Spain. 6 Hospital Clínico Universitario, Valencia. Biomedical Research Institute INCLIVA, Universidad de Valencia, Valencia; Spain. 7 Instituto Valenciano de Oncología, Valencia; Spain. 8 Servicio de Oncología Médica, Hospital Clínico San Carlos, Instituto de Investigación Sanitaria del Hospital Clínico San Carlos (IdISSC), Madrid, Spain; 9 Hospital Universitario La Fe, Valencia; Spain. 10 Complejo Hospitalario Virgen de la Victoria, Málaga; Spain. 11 Hospital Universitario Lozano Blesa, Zaragoza; Spain. 12 Hospital de Basurto, Bilbao; Spain. 13 Complejo Hospitalario Juan Canalejo, A Coruña; Spain. 14 Complejo Hospitalario de Albacete, Albacete; Spain. 15 Hospital Universitario de Canarias, Sta Cruz de Tenerife; Spain. 16 Hospital Universitario de Lleida Arnau de Vilanova, Lleida; Spain. 17 Hospital General Universitario Miguel Servet, Zaragoza; Spain. 18 Instituto Oncológico de Guipúzcoa, San Sebastián; Spain. 19 Complejo Hospitalario Reina Sofía, Córdoba; Spain. 20 Hospital Universitario Marqués de Valdecilla, Santander; Spain. On behalf of GEICAM, the Spanish Breast Cancer Group.

Supplementary Table 1. Restricted multivariate analysis for overall survival for the low risk *de novo* MBC patients from *El Álamo* registry.

Variable	N	HR	95%CI	p-value
**Surgery**				0.103
Yes	172	0.742	(0.518 – 1.063)	
No	79	Ref.		
**Metastatic location**				<0.001
Visceral	66	Ref.		
Bone (if non visceral)	146	0.587	(0.401 – 0.860)	0.006
Soft tissue (if non bone non visceral)	39	0.284	(0.161 – 0.504)	<0.001
**Histological type**				0.042
Ductal	165	Ref.		
Other	35	1.638	(1.025 – 2.617)	0.039
NA	51	0.791	(0.489 – 1.278)	0.338
**Histological Grade**				0.014
G I-II	86	Ref.		
G III	44	1.929	(1.191 – 3.125)	0.008
NA	121	1.022	(0.662 – 1.577)	0.923
**Hormone Receptor status**				0.272
Positive	110	Ref.		
Negative	40	1.389	(0.829 – 2.330)	0.212
NA	101	1.375	(0.882 – 2.142)	0.160
***El Álamo* cohort**				0.040
El Álamo I	59	Ref.		
El Álamo II	121	0.623	(0.415 – 0.938)	0.023
El Álamo III	71	0.561	(0.341 – 0.922)	0.023

Abbreviations: MBC, metastatic breast cancer; HR, hazard ratio; CI: confidence interval; G, grade; NA not-assessed.
